# A Lactic Acid Bacteria Consortium Impacted the Content of Casein-Derived Biopeptides in Dried Fresh Cheese

**DOI:** 10.3390/molecules27010160

**Published:** 2021-12-28

**Authors:** Jasna Novak, Katarina Butorac, Andreja Leboš Pavunc, Martina Banić, Ana Butorac, Adriana Lepur, Nada Oršolić, Katarina Tonković, Krešo Bendelja, Nina Čuljak, Marija Lovrić, Jagoda Šušković, Blaženka Kos

**Affiliations:** 1 Laboratory for Antibiotic, Enzyme, Probiotic and Starter Cultures Technology, Faculty of Food Technology and Biotechnology, University of Zagreb, Pierottijeva 6, 10000 Zagreb, Croatia; jasna.novak@pbf.unizg.hr (J.N.); kzoric@pbf.hr (K.B.); alebos@pbf.hr (A.L.P.); mmarijanovic@pbf.hr (M.B.); nculjak1@pbf.hr (N.Č.); jsusko@pbf.hr (J.Š.); 2 BICRO Biocentre Ltd., Borongajska cesta 83H, 10000 Zagreb, Croatia; ana.butorac@biocentre.hr (A.B.); adriana.lepur@biocentre.hr (A.L.); marija.lovric@biocentre.hr (M.L.); 3 Department of Animal Physiology, Faculty of Science, University of Zagreb, Rooseveltov trg 6, 10000 Zagreb, Croatia; nada.orsolic@biol.pmf.hr; 4 Probiotik Ltd., Grada Gospića 3, 10000 Zagreb, Croatia; katarina.tonkovic@probiotik.hr; 5 Centre for Research and Knowledge Transfer in Biotechnology, University of Zagreb, Rockefellerova 10, 10000 Zagreb, Croatia; kreso.bendelja@unizg.hr

**Keywords:** biopeptides, cheese, probiotic, proteinases, *Lactobacillus*, *Enterococcus*, starter culture

## Abstract

This study aimed to define a consortium of lactic acid bacteria (LAB) that will bring added value to dried fresh cheese through specific probiotic properties and the synthesis of bioactive peptides (biopeptides). The designed LAB consortium consisted of three *Lactobacillus* strains: S-layer carrying *Levilactobacillus brevis* D6, exopolysaccharides producing *Limosilactobacillus fermentum* D12 and plantaricin expressing *Lactiplantibacillus plantarum* D13, and one *Enterococcus* strain, *Enterococcus faecium* ZGZA7-10. Chosen autochthonous LAB strains exhibited efficient adherence to the Caco-2 cell line and impacted faecal microbiota biodiversity. The cheese produced by the LAB consortium showed better physicochemical, textural and sensory properties than the cheese produced by a commercial starter culture. Liquid chromatography coupled with matrix-assisted laser desorption/ionization-time of flight tandem mass spectrometry (LC-MALDI-TOF/TOF) showed the presence of 18 specific biopeptides in dried fresh cheeses. Their identification and relative quantification was confirmed by liquid chromatography-tandem mass spectrometry (LC-MS/MS) using multiple reaction monitoring (MRM). The results also showed that their synthesis resulted mainly from β-casein and also α-S1 casein degradation by proteolytic activities of the LAB consortium. The designed LAB consortium enhanced the functional value of the final product through impact on biopeptide concentrations and specific probiotic properties.

## 1. Introduction

Consumption of fermented (dairy) foods alleviates the negative consequences of modern Western-type diets, which may result in alteration of gut microbiome composition. Therefore, due to the health benefits and the growing appreciation of fermented dairy foods, the aim of this study was to design a consortium of lactic acid bacteria (LAB) that could be applied as a mixed starter culture during cheese fermentation. Additionally, since some LAB strains have great potential as producers of bioactive peptides from casein, we were interested in their proteolytic potential. Specific bioactive peptides released during cheese fermentation by LAB appear to mediate physiological processes associated with beneficial health effects [1,2,3,4]. Their amino acid sequences are encrypted within caseins and whey proteins and can be released by proteolysis. Caseins can be hydrolysed by digestive enzymes such as pepsin in the gastrointestinal tract (GIT) or by microbial or plant proteinases [5,6]. Research has reported the spectrum of biological activities of casein-derived peptides with effects on the gastrointestinal, immune, cardiovascular and nervous systems [2,7,8]. Recently, Popović et al. [3] proved that yogurt fermented by autochthonous *Streptococcus thermophilus* and *L. bulgaricus* strains positively impacted gut barrier by cross-linking key processes associated with epithelial homeostasis. Aguilar-Toalá et al. [9] demonstrated the multifunctional roles of peptides accumulated in fermented milk by the action of *L. plantarum* strains isolated from semi-soft cheeses.

Previously, based on in vitro screening of a large number of autochthonous LAB strains isolated from artisanal fresh and dried fresh cheeses, we selected LAB strains with specific properties, characterised as probiotic: an S-layer carrying *Levilactobacillus brevis* D6 [10,11], an exopolysaccharides producer *Limosilactobacillus fermentum* D12 [12], a plantaricin expressing *Lactiplantibacillus plantarum* D13 [10,13] and *Enterococcus faecium* ZGZA7-10. Interestingly, *Enterococcus* species encompassed 29.8% of LAB strains isolated from Croatian fresh soft cheeses and were found to contribute significantly to the aroma and texture-specific features of these artisanal cheeses [14,15]. We especially sought to characterise the impact of selected LAB strains on faecal microbiota of rats and proteolytic activities during dried fresh cheese production that may increase the concentration of the putative biopeptides and finally result in the additional functional value of the end-product. Mass spectrometry of casein hydrolysate peptides, potentially generated with selected LAB strains during the fermentation, was applied for the identification of the peptide sequences. Furthermore, proteomic analysis was used for the detection of the proteinase complex responsible for casein degradation.

## 2. Results

### 2.1. Techno-Functional Features of the LAB Consortium

We previously analysed the proteolytic potential of over 100 different LAB strains of *Lactobacillus, Lactococcus*, and *Enterococcus* based on the appearance of transparent zones on skimmed milk agar plates [16]. Comparison of the activities between the examined *Lactobacillus* strains showed that *L. brevis* D6, *L. fermentum* D12, and *L. plantarum* D13 were effective in casein proteolysis [16]. Their potential to degrade casein was further assessed by determining protease activities by Anson’s method (Table 1a). Strain *E. faecium* ZGZA7-10 showed the highest protease activity, followed by *Lactobacillus* strains D6, D12 and D13, based on the measurement of released tyrosine. Casein degradation products were also determined by SDS-PAGE after incubation with culture supernatants of LAB strains grown overnight in skimmed milk (data not shown). According to Hebert et al. [17], the proteolytic properties of LAB strains correlate to their fast milk-coagulating (Fmc) phenotype.

The quantification of lactose, and the resulting metabolites, lactate, acetate and diacetyl, after incubation of strains *L. brevis* D6, *L. fermentum* D12, *L. plantarum* D13 and *E. faecium* ZGZA7-10 in skimmed milk, was performed by chromatography approaches (Table 1a). Among the four strains, the highest lactate concentration was determined for *E. faecium* strain ZGZA7-10, while the highest concentration of acetate was determined for *L. plantarum* D13, after growth in skimmed milk. The lactose concentrations in all skimmed milk samples ranged from 46.50 ± 0.50 to 51 ± 1.0 mg/mL, with the lowest determined for the ZGZA7-10 strain. Diacetyl was not detected in any sample of skimmed milk after cultivation of the strains as revealed by the FID GC method (Table 1a). The threshold for diacetyl has been reported to range from 0.001 to 0.550 ppm, depending on the food matrix and the method of assessment, with the lowest value being reported for cheese [18]. Additionally, *E. faecium* ZGZA7-10 did not show any haemolytic activity (Table 1a) and was susceptible to antibiotics (data not shown), which is a prerequisite to be applied as a starter culture according to EFSA regulations [19].

To assess their applicability in cheese fermentation, selected strains were tested for the capacity to decrease pH, lactic acid production and tolerance to different NaCl concentrations during the growth in skimmed milk (Table 1b). LAB strains acidified skimmed milk and efficiently produced lactic acid, especially *L. plantarum* D13(Table 1b). The examined LAB strains tolerated NaCl addition, but the acidification rate and lactic acid production decreased with increasing NaCl concentrations, as expected. Strain ZGZA7-10 showed the potential to produce lactic acid in the presence of 2% and 4% NaCl with the lowest dropout rate compared to other strains (Table 1b). It is obvious that increased salt concentrations inhibited LAB growth and limited their ability to coagulate milk (data not shown). Previously, examined LAB strains also showed the fastest acidification rate and the highest coagulation capacities after overnight incubation in skimmed milk s [16].

### 2.2. Whole-Genome Sequencing and Analysis of Proteolytic System by LC-MALDI-TOF/TOF

**Figure 1:**
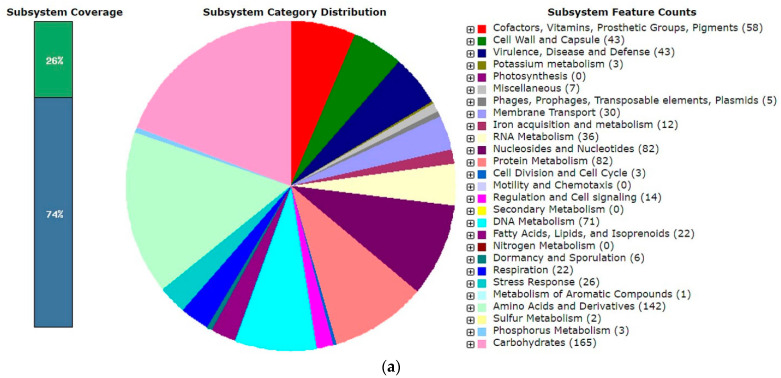

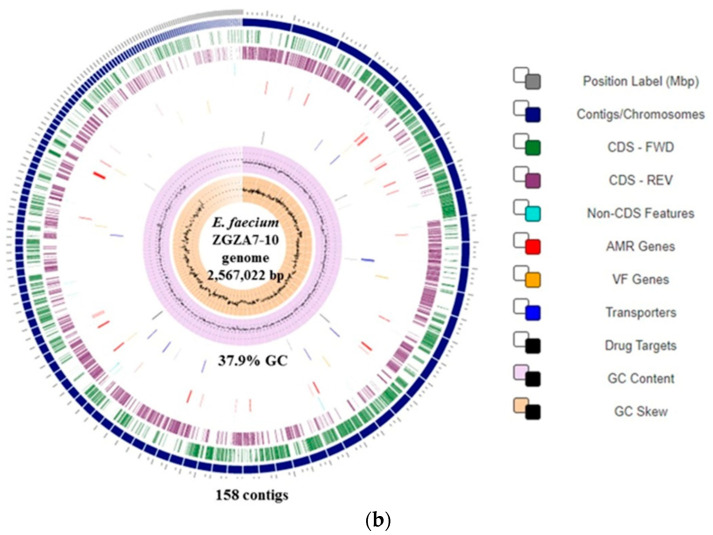
Functional annotation of *E. faecium* ZGZA7-10: (**a**) by RAST; (**b**) circular genome map generated with PATRIC. From outer to inner rings are contigs, coding sequence (CDS) on the forward strand, CDS on the reverse strand, RNA genes, CDS with homology to known antimicrobial resistance genes, CDS with homology to known transporters, CDS with homology to known drug targets, GC content, and GC skew.

The whole-genome sequences (WGS) of the *Lactobacillus* strains of the designed LAB consortium have been analysed previously [10,12,13] and are available (GenBank Accession number: D6 strain LQNG00000000.1; D13 strain NIGG00000000 and D12 strain RHMA01000000), while the analysis of the WGS of strain ZGZA7-10 is available under GenBank Accession number JAJAGE000000000 and Bioproject Accession number PRJNA388578. Although previous 16S RNA sequencing and BioNumerics™ 7.5 software-generated AFLP^TM^ DNA fingerprints suggested that the strain ZGZA7-10 is *Lactococcus lactis* subsp. *lactis* (data not shown), WGS analysis revealed that this strain belongs to *Enterococcus faecium*. Initial assembly of the pair-end Illumina sequencing reads resulted in 158 scaffolds (Figure 1b). The ordered scaffolds had a length of 2,567,022 bp and a GC content of 37.9%. Annotation with RAST identified 2605 coding DNA sequences (CDS) and 25 RNA sequences (Figure 1a). From the total number of proteins encoded by the ZGZA7-10 genome, 74% and 26% could or could not be assigned in subsystem categories, respectively. Focusing on the proteolytic activity of the strain ZGZA7-10, preliminary analysis identified five aminopeptidases in the subsystem counts for aminopeptidases and seven in subsystem counts for protein degradation. Additionally, when checking protein encoding genes (PEG) assigned to the subsystem category membrane transport, a set of oligopeptide transporters ABC was detected. Since the ZGZA7-10 strain expresses the Fmc phenotype, hydrolyses casein, and its genome possesses several genes related to protease activities, we were interested in characterising its putative proteolytic system. LC-MALDI-TOF/TOF analysis of total cellular proteins was performed to identify enzymes associated with proteolytic activities. The proteins coding for enzymes that are possibly involved in casein hydrolysis were detected, and those also coding for peptide transporters or peptidases (Supplementary Materials Table S1a,b). Preliminary analysis of the proteome revealed that the ZGZA7-10 strain expresses enzymes of the caseinolytic protease (Clp) complex.

### 2.3. The Capacity of the LAB Consortium for Transient Colonisation

The potential of probiotic bacteria to colonise GIT and alter the composition of the gut microbiota is thought to be a key mechanism for their functional activities. Therefore, our interest was to assess the potential of the LAB consortium to withstand GIT transit when applied as mixed culture. The survival of the applied strains in GIT conditions in vitro has been previously demonstrated [10,12,13]. Here, we used two different experimental approaches: monitoring of the adhesion of the individual LAB strains to intestinal epithelial cells, and microbial profiling to assess their influence on the faecal microbiota when applied as a consortium to rats.

**Figure 2:**
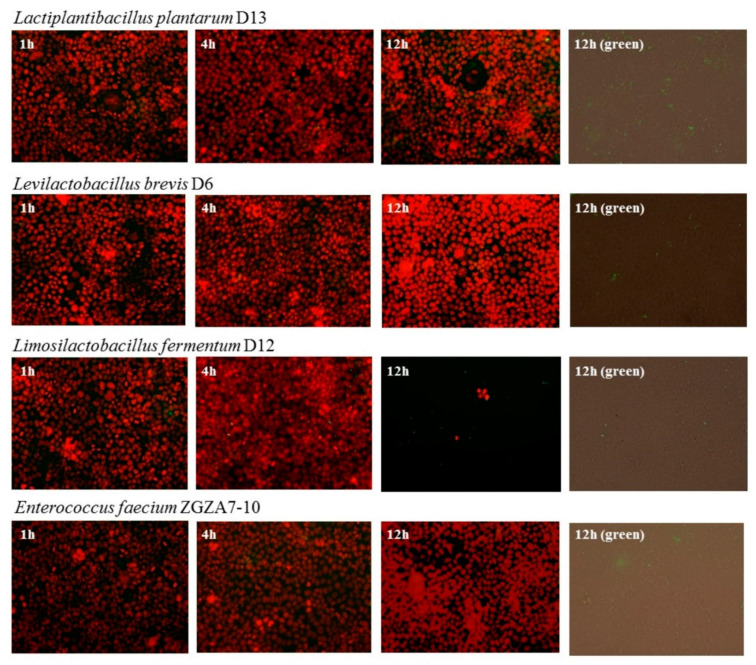
Fluorescence microscopy images of adhesion of individual strains of the LAB consortium to Caco-2 cell monolayer: D6, D12, D13 and ZGZA7-10 bacterial cells. Fluorescence overlays of Caco-2 cell nuclei (red fluorescence) with adhered LAB cells (green fluorescence) at a seeding density of multiplicity of infection (MOI) = 50, after 1, 4 and 12 h of incubation (overlay, and only green fluorescence) at 20× magnification.

The adhesion assays were performed using the Caco-2 cell lines. Adherence to the Caco-2 cell line was previously determined for *L. brevis* D6, *L. plantarum* D13 and *L. fermentum* D12 [10,12,13]. In this work, the adherence of each of four strain of LAB consortium strains was observed under the same conditions using fluorescent vital staining. Adhesion of D6, D12, D13 and ZGZA7-10 strains to the Caco-2 cell monolayer at MOI = 50 after 1, 4 and 12 h of incubation is shown (Figure 2). *L. brevis* D6 and *L. fermentum* D12 were the most adhesive when compared among the examined LAB strains.

**Figure 3:**
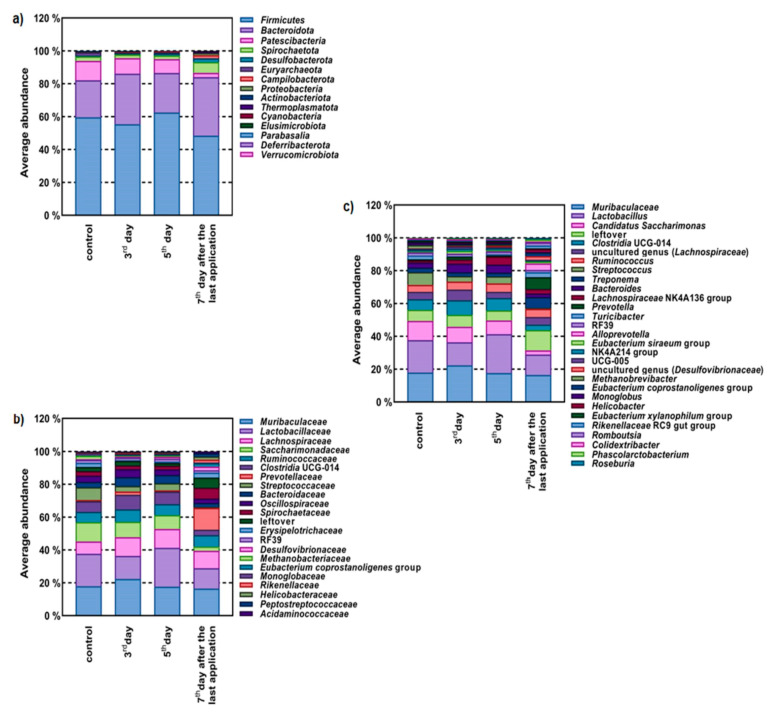
Influence of LAB consortium (D6, D12, D13 and ZGZA7-10 strains) on the composition of the faecal microbiome of rats determined through relative abundance of OTU at: (**a**) phylum; (**b**) family; and (**c**) genus level. Legend: Control—before LAB consortium application; 3rd and 5th day of LAB consortium application; 7th day after the last application of LAB consortium.

Furthermore, we monitored the in vivo influence on faecal microbiome profiling after application of the LAB consortium in experimental rats (Figure 3). The dominant phyla were *Firmicutes* and *Bacteroidota*, representing more than 80% of total gut microbiota (Figure 3a). Results showed that these phyla were strongly represented by the *Muribaculaceae* and *Lactobacillaceae* families, and bacteria genera such as *Lactobacillus* spp. The most abundant in all groups were *Muribaculaceae, Lactobacillaceae, Lachnospiraceae* and *Ruminococcaceae* (Figure 3b). Following administration of the LAB consortium, an increase in faecal microbiota biodiversity was observed (Figure 3b). The administration of the LAB consortium resulted in the appearance of the phyla *Thermoplasmatota*, *Cyanobateria*, *Elusimicrobiota*, *Verrucomicrobiota* and *Parabasalia*, leading to a three-fold increase in the number of phyla present in the rat gut microbiota (Figure 3a). At the family level (Figure 3b), the most striking change was more than a 17-fold increase in the abundance of *Prevotellaceae*, producers of short-chain fatty acids (SCFAs). Additionally, the LAB consortium impacted the enrichment of *Lachnospiraceae* and *Ruminococcaceae*, important butyrate producers residing in the intestinal microbiota. At the genus level, administration of the LAB consortium resulted in a significant shift in the abundance of several genera and a transient increase in the abundance of bacteria belonging to the genus *Lactobacillus* (Figure 3c). Most prodigious, however, was the more than 58-fold increase in abundance of *Alloprevotella* and the more than 10-fold increase in abundance of *Prevotella*, both potent SCFA-producing genera. In addition, the LAB consortium caused a 13-fold decrease in abundance of common rat pathogens belonging to the genus *Streptococcus*.

### 2.4. Application of a Designed LAB Consortium in Cheese Fermentation

The designed LAB consortium was applied for the fermentation of dried fresh cheese. In addition to evaluating the potential of the LAB consortium as the functional starter culture, another aim was to examine possibility of the synthesis of bioactive peptides by the LAB consortium. This would confirm proteolytic activity as an additional functional property of the LAB consortium. We compared the properties of dried fresh cheese after using a defined LAB consortium to those fermented by the commercial mesophilic starter DSM CT-203 used in the industrial production of fresh (unripened) cheeses. The pH, dry matter, contents of fat, lactose, yield and syneresis of cheese samples are presented in Table 2.

The dried fresh cheese made with the LAB consortium and dried fresh cheese made with starter DSM CT-203 culture did not differ significantly in pH values, dry matter and syneresis. Cheese made with the LAB consortium had lower fat (19.06 ± 0.16%, *w*/*w*) and lactose (4.24 ± 0.21%, *w*/*w*) content compared to the values determined in the control cheese (26.57 ± 0.24%, *w*/*w* and 5.82 ± 0.25%, *w*/*w*). Both types of cheeses were devoid of *Listeria* or *Salmonella* contamination as determined by microbiological analysis (Table 2). The results of the sensory analysis indicated that the cheese produced by using the LAB consortium was of uniform quality with higher creaminess than control cheese, as well as a small number of irregular pots of uniform size, a pleasant lactic acid smell and a moderately salty taste. In contrast, control cheese was found to have a weaker lactic acid associated odour and a slightly sour taste, which was less appreciable, compared to the cheese produced by the LAB consortium. The separated whey of the LAB consortium cheese showed better test scores regarding precipitate and taste than control whey (Table 3). The preliminary analysis of microbial communities of dried fresh cheese fermented with LAB consortium through metagenomic 16S rRNA sequencing confirmed the abundance of *L. brevis* (1.22%), *L. fermentum* (1.01%) and *Enterococcus* spp. (6.97%).

Since the LAB consortium used in fermentation showed a positive impact on the physico-chemical, textural, and sensory properties of dried fresh cheese, we studied whether their application could favour the release of biopeptides to achieve the additional functional properties of produced dried fresh cheese. According to the results of LC-MALDI-TOF/TOF, various peptides which may result from the protease activities of the LAB consortium, were identified (Table 4). Furthermore, we applied the LC-MRM-MS method for targeted analysis of peptides released in skimmed milk inoculated with single strains of the LAB consortium as well as in two produced types of cheeses and their separated whey. Based on this initial discovery LC-MALDI-TOF/TOF experiment, a total of 18 target peptides from α-S2-CN and β-CN were selected for targeted analysis (Table 4a). The proteolytic potential of each of the LAB consortium strains was confirmed by the detection of casein-derived peptides after cultivation in skimmed milk (Table 4). Moreover, 15 peptides were detected in dried fresh cheese fermented by the LAB consortium. Of these, 11 biopeptides were detected in skimmed milk fermented by individual strains of the LAB consortium, as well as in cheese and whey produced by the LAB consortium. A search of MBPDB revealed that detected peptides might exert antimicrobial, anti-inflammatory, ACE-inhibitory and antioxidant activities. Interestingly, HQPHQPLPPTVMFPPQ (β-CN) was detected exclusively in the skimmed milk fermented by the D12 strain, as well as in cheese samples produced by the LAB consortium and the resulting whey (Table 4b). In the dried fresh cheese fermented with the LAB consortium, the β-CN peptides WMHQPHQPLPPT, LVYPFPGPIHNSLPQN, VYPFPGPIPN, QEPVLGPVRGPFPIIV and YQEPVLGPVRGPFPIIV were more abundant, while the three peptides TKVIPYVRYL, LVYPFPGPIHNSLPQ and LYQEPVLGPVRGPFPIIV were more abundant in the control cheese (Table 3b).

## 3. Discussion

Autochthonous cheeses are potential functional foods attractive as carriers for the delivery of probiotic bacteria. The aim here was to design a consortium of LAB, with specific probiotic properties of the strains and apply them as a functional starter culture in cheese making. Since proteolysis is crucial for cheese flavour development, as well as for biopeptide accumulation, we studied the proteolytic potential of a representative set of autochthonous LAB strains. In the first stage, we screened more than 100 LAB strains for proteolytic activities, including genera frequently used as dairy starter cultures [16]. In this study, LAB strains with the highest caseinolytic activity were further characterized. This included *L. brevis* D6, *L. fermentum* D12 and *L. plantarum* D13 previously identified by WGS and with characterised probiotic specificities [10,12,20]. We previously demonstrated that the EPS producing D12 strain inhibited enteropathogenic bacteria, survived simulated GIT conditions and adhered to Caco-2 cells in vitro [12]. Moreover, the produced EPS positively influenced the capacity of the D12 strain to survive during freeze-drying and to adhere to extracellular matrix (ECM) proteins. While S-layer-carrying *L. brevis* D6 has advantageous probiotic potential related to the S-layer protein’s role, particularly in protection of rigorous GIT conditions and adhesion to intestinal cells lines with potential immunomodulatory capacity, plantaricin expressing *L. plantarum* D13 can additionally contribute to the competitive exclusion of pathogens through the antimicrobial and adhesive properties [10,12]. Strain *E. faecium* ZGZA7-10, which showed the highest protease activity among the chosen consortium of LAB strains, was further characterised through genome sequencing determined using a WGS assembly approach, and was found to be safe regarding non-haemolytic and antibiotic susceptibility features [19]. The role of *E. faecium* strains during cheese fermentations has not been yet fully elucidated due to the low interest in enterococci resulting from the association of certain clinical strains with nosocomial infections. Recently Papadimitriou et al. [21] emphasised important technological properties, including proteolytic potential and bacteriocin production of *E. faecalis* PK23, isolated from autochthonous white brine cheese, which is, like *E. faecium* strains, often neglected due to safety concerns. The *E. faecium* ZGZA7-10 strain was previously isolated from fresh soft cheese from the Zagorje region in Croatia where, as well as in Southern Europe, artisanal cheeses may contain up to 10^7^ CFU/g enterococci at the end of cheese ripening, thereby contributing to their typical sensory properties [14,15,22]. This is consistent with studies in which *E. faecium* and *E. faecalis* were reported as the most prevalent species of *Enterococcus* genus among raw milk and dairy products microbiota [14,15,23,24,25]. Leboš Pavunc and coworkers [14] reported about efficient production of fresh cheese using a combination of commercial starter culture and autochthonous strains *E. faecium* A7 and *L. fermentum* A8. This resulted in improved sensorial properties, more similar to those of spontaneously fermented fresh cheese than to those of cheese produced with only commercial starter culture. *Enterococcus* strains represent a significant part of the autochthonous microbiota of Croatian cheeses, with *Enterococcus faecalis* and *E. faecium* being the predominant species [14,15]. *Enterococcus* strains are usually present as a nonstarter LAB that contribute to the microbial safety of the cheese, as some strains show anti-listerial activity. None of the cheese samples was positive for *L. monocytogenes* and *Salmonella* spp., which are common food-borne pathogens screened in dairy products as indicators of safety. Enterococci also display efficient proteolytic activity, which contributes to the sensory characteristics of fermented dairy products [23].

An important aspect of selecting LAB strains for use as starter cultures is performance testing, in which growth rate, acid production and adaptation to salt are assessed [25]. In this research, fast acidifiers of skimmed milk were selected. This was confirmed by the determination of lactic and acetic acid by HPLC-UV/DAD chromatography which has been successfully applied for the quantitative identification of LAB metabolites [26]. As expected, selected strains that are obligatory or facultative heterofermentative LAB produced both lactic acid and acetic acid by exploiting lactose. Besides determining organoleptic characteristics, these metabolites act as biopreservatives that prevent the growth of contaminants, contributing to product safety and quality. Selected strains rapidly synthesized lactic acid and efficiently coagulated milk overnight when used as a 1% inoculum. Moreover, it appears that, although their metabolic activity was affected they still adapted well to exposure to the increasing salt concentrations found during cheese manufacture. Diacetyl production was not observed for any strain. According to Clark and Winter [18], the content of diacetyl in a diversity of cheeses was in the range of 0.02–13.68 ppm, which contributes to the typical butter-like flavour of the cheeses, while larger amounts of diacetyl are associated with flavour defects. Overall, the results imply their potential as promising dairy starter cultures.

To demonstrate further techno-functional characteristics, we focused on the capacity of the LAB strains to transiently colonize GIT. Since four species showed potential to survive GIT passage in vitro, to further study their colonisation capacity we observed adhesion to a Caco-2 cell monolayer by fluorescence microscopy and thereafter monitored their survival when administrated as a mix culture in vivo by microbiome profiling. *L. brevis* D6 and *L. fermentum* D12 were the most adhesive between the four strains. Their competition among intestinal microbiota was detected by rat faecal microbiome profiling after LAB consortium treatment. The largest share in the faecal microbiome of each examined rat consisted of bacteria belonging to the phyla *Firmicutes* and *Bacteroidota*. Accordingly, an increased abundance of the family *Muribaculaceae* belonging to the *Bacteroidota* phylum was observed. This is consistent with a recent study by Bowerman et al. [27] which reported that the *Muribaculaceae* family is often observed as fluctuating in abundance in the microbiota of laboratory mice, and can change from below detection to representing more than half of the bacterial community. Our study showed that the intestinal microbiota composition of the rats treated with the LAB consortium markedly differed from their controls. In addition, administration of the LAB consortium resulted in an increase in *Prevotellaceae* and *Lachnospiraceae*, important producers of SCFAs. The potential of probiotic strains to alter the intestinal microbiota was also observed by Lv et al. [28], who administered a combination of *Bifidobacterium*, *Lactobacillus*, *Enterococcus*, and *Bacillus* strains to immunocompromised rats, as evidenced by faecal microbiome profiling. Overall, our results indicate that the designed consortium of LAB strains can alter the intestinal microbiota, which could be of value for future probiotic-targeted analysis of these LAB strains.

As mentioned, the design consortium included three representatives of *Lactobacillus* and one strain of *Enterococcus*. All four strains showed techno-functional characteristics, based on their caseinolytic and metabolic activities in skimmed milk, which are supported with the previously characterised specific probiotic potential of three individual *Lactobacillus* strains, with specific features such as S-layer proteins, plantaricin and EPS synthesis [10,12,13]. Therefore, the LAB consortium was applied as a functional starter culture for cheese production. Cheese made with the LAB consortium had lower pH values than cheese made with the commercial DSM CT-203 culture. The acidity of the cheese in the current study was generally lower than the values (4.54–5.20) for soft cheese reported by Bekele et al. [29]. The LAB consortium surpassed DSM CT-203 in terms of acidification rate, cheese yield, fat content, and lactose depletion. The addition of a LAB consortium to milk facilitated its coagulation by increasing the lactic acid content and improving curd firmness. This could be attributed to the Fmc phenotype of the LAB consortium. Furthermore, the cheese fermented by the LAB consortium had better consumer preference scores for the taste and odour of cheese and whey compared to the control. The differences in preference test scores in this study, particularly in the taste and odour of the cheese samples, might be attributed to the specific properties of the different LAB strains applied. Namely, microbiome analysis of produced cheese samples by 16S amplicon sequencing revealed the abundance of *Lactobacillus*, with a representative share of *L. brevis, L. fermentum* and *Enterococcus* strains, suggesting their adaptation to the cheese microenvironment. It can be hypothesised that creaminess could be also associated with in situ EPS synthesis by *L. fermentum* D12 [12]. According to Surber et al. [30] LAB producing ropy EPS may contribute to the improved texture and syneresis of cream cheese. Namely, EPS synthesising LAB have been applied for thickening and stabilizing fermented dairy products, resulting with higher microbiological quality and reduced production costs [30].

The main metabolic pathway of LAB involved in cheese flavour formation, in addition to lactose and citrate metabolism, is proteolysis [31]. The proteolysis performed by the starter culture results in the accumulation of peptides and amino acids [32]. Caseinolytic enzymes of LAB may even act as milk coagulants but have especially been studied for the possibility of biopeptide synthesis [2,33]. Here, we used MS-based peptidomics for peptide detection in dried fresh cheeses and separated whey. The hydrolysis of an α-CN and β-CN in milk samples was detected by quantitative LC-MRM-MS analysis of their target peptides. The peptide profile of dried fresh cheese is dependent on the mix starter culture used. We identified 15 casein peptides in the dried fresh cheese fermented by the LAB consortium. Of those, 10 casein peptides were significantly higher abundant, with two being specific (HQPHQPLPPTVMFPPQ and LVYPFPGPIHNSLPQN) compared to the control cheese. Among four β-CN peptides, WMHQPHQPLPPT shows anti-inflammatory capacity, LVYPFPGPIHNSLPQN [30] and VYPFPGPIPN [31] exert ACE-inhibitory activity, while YQEPVLGPVRGPFPIIV, produced by all of the consortium LAB strains in monoculture, exerts several beneficial effects, including ACE-inhibitory, immunomodulatory, antithrombotic and antimicrobial activities [33,34,35]. The ACE-inhibitory peptide YQEPVLGPVRGPFPIIV has been reported in several kinds of cheese produced in different regions [35]. Another casein-derived peptide, WMHQPHQPLPPT was also significantly more abundant in cheese fermented by the LAB consortium compared to the control. According to Adams et al. [36] the same peptide has been identified as part of a larger bioactive fraction collected from the supernatant of fermented milk. The results support the hypothesis that the designed LAB consortium may affect the biopeptides concentration during milk fermentation. Considering previously characterised specific probiotic properties of the contained LAB strains, their influence on rat microbiota biodiversity, as well as biopeptide synthesis, improved the functional value of the final fermented dairy product, and dried fresh cheese was achieved.

## 4. Materials and Methods

### 4.1. Bacterial Strains

Four LAB strains isolated from autochthonous Croatian cheeses, three of them previously described (S-layer producing *Levilactobacillus brevis* D6, EPS producing *Limosilactobacillus fermentum* D12 and plantaricin expressing *Lactiplantibacillus plantarum* D13) with sequenced genomes [10,12,37] and *E. faecium* ZGZA7-10, were used in this study. *Lactobacillus* stock cultures were maintained at −80 °C in MRS broth (BD Difco, Detroit, MI, USA) and *Enterococcus* in M17 broth (Biolife, Milan, Italy) with 15% (*v*/*v*) glycerol (Sigma-Aldrich, Saint Louis, MO, USA). Working cultures were prepared from frozen stocks by two sequential transfers in MRS or M17 broth and incubated at 37 °C overnight. The overnight LAB culture was harvested by centrifugation at 4000× *g* for 10 min. The cell pellet was suspended in 0.9% NaCl (*w*/*v*) (Kemika, Zagreb, Croatia) and the optical density at 620 nm (OD_620_) was determined. The resulting pellet was suspended in 0.9% NaCl to obtain a final concentration of 10^10^ CFU/mL. The final bacterial suspension was monitored daily by pour plate method on agar plates before administered to the rats.

### 4.2. WGS of E. Faecium ZGZA7-10

Genomic DNA of ZGZA7-10 strain was extracted using a Maxwell^®^ DNA Cell kit in a Maxwell^®^ 16 Research System instrument (Maxwell, Promega, Madison, WI, USA). Sequencing was performed on an Illumina MiSeq 2500 (Illumina, San Diego, CA, USA) at IGA Technology Services (IGA Technology Services Srl, Udine, Italy) using a paired-end approach, as described in Banić et al. [31]. Contigs were classified as belonging to *E. faecium* ZGZA7-10 when obtaining the best BLASTn v2.2.27 hit (45) in the NCBI nt database, where the whole genome sequence was submitted. The genome of ZGZA7-10 was uploaded to the web annotation service Rapid Annotations using Subsystems Technology (RAST; http://rast.nmpdr.org/rast.cgi accessed on: 25 November 2021) for automated annotation of sequenced genes, followed by manual scanning. The Whole Genome Shotgun project has been deposited at DDBJ/ENA/GenBank under the accession number JAJAGE000000000 (BioProject PRJNA388578, Biosample SAMN22155556). This version of the project (01) consists of sequences JAJAGE010000001-JAJAGE010000158. A circular map of the ZGZA7-10 genome was created using the PATRIC.

### 4.3. Determination of Fermentation Parameters during LAB Growth in Skimmed Milk

*L. brevis* D6, *L. fermentum* D12, *L. plantarum* D13, and *E. faecium* ZGZA7-10 were inoculated into skimmed milk (2%) (Sigma-Aldrich, Merck, St. Louis, MO, USA) or in skimmed milk with added 2.0, 4.0 and 6.5% (*w*/*v*) of NaCl and incubated for 48 h at 37 °C. During cultivation, lactic acid production, pH value, and the bacterial counts were monitored. Proteolytic activity of examined strains after 4 h of incubation in 0.65% (*w*/*v*) casein was quantified according to Anson’s method as described by Beganović et al. [38]. Quantification of lactose, lactate and acetate after overnight growth of particular LAB strains in skimmed milk was performed by liquid chromatography (HPLC-UV/VIS-DAD) while diacetyl was determined by gas chromatography (GC-FID). D-lactose monohydrate (Sigma-Aldrich, Merck, St. Louis, MO, USA), anhydrous sodium acetate (Sigma-Aldrich, Merck, St. Louis, MO, USA), sodium-d,l-lactate (Sigma-Aldrich, Merck, St. Louis, MO, USA), and diacetyl (Sigma-Aldrich, Merck, St. Louis, MO, USA), were used as analytical standards. The quantification limit of the HPLC/UV/VIS (DAD) method is 0.00625 mg/mL for acetate and 0.00625 mg/mL for lactate. The quantification limit for the determination of diacetyl by the GC-FID method is 0.1 mg/mL.

### 4.4. Adhesion to Caco-2 Cells

Adhesion experiments were performed as described previously with slight modifications [39]. Monolayers of Caco-2 cell lines were seeded on 96-well plates (Greiner Bio-One, Kremsmünster, Austria) in DMEM/F12 (Dulbecco’s modified Eagle Medium, Capricorn Scientific GmbH, Ebsdorfergrund, Germany) + l-glutamine (Merck, Darmstadt, Germany) + 10% FBS (Fetal Bovine Serum, Gibco, by Life Technologies, Carlsbad, CA, USA) and washed two times with DMEM. The cells were marked with Incucyte Nuclight Rapid Red dye (PHI AB, Boston, MA, USA). D6, D12, D13 and ZGZA7-10 strains were grown overnight at 37 °C in MRS broth. The bacterial culture was centrifuged at 5000× *g* for 5 min, and the pellet was washed two times in sterile PBS (phosphate-buffered saline, Gibco, by Life Technologies, Carlsbad, CA, USA). The pellet was adjusted to a cell concentration of 2 × 10^8^ cells/mL. The bacterial cells of examined LAB strains were resuspended in 5 mM Viafluor 488 (Biotium, Fremont, CA, USA), incubated for 15 min at 37 °C, resuspended in DMEM and added to Caco-2 monolayers and incubated at 37 °C for 1 h with gentle shaking. After incubation, the cell monolayers were washed gently thrice with PBS and fixed with 4% paraformaldehyde (Gibco, by Life Technologies, Carlsbad, CA, USA) in PBS. Fluorescence microscopy images of Caco-2 cells and bacterial cells were acquired using an EVOS FLc Cell Imager (Thermo Fisher Scientific, Waltham, MA, USA). Fluorescence overlays of Caco-2 cell nuclei (red fluorescence) with adhered LAB cells (green fluorescence) at a MOI = 50, after 1, 4 and 12 h of incubation were detected at 20× magnification.

### 4.5. Rat Study Design

#### 4.5.1. Experimental Animals

Three-month-old male highly inbred Y59 strain rats (*n* = 12) were bred and maintained in the Department of Animal Physiology, Faculty of Science, University of Zagreb (http://www.informatics.jax.org/external/festing/rat/docs/Y59.shtml accessed on 15 October 2021). The animals were maintained under a 12/12 h light–dark cycle with free access to food and water and standard housing conditions. The ambient temperature was 23–25 °C and the relative humidity 60%. Rats were fed a standard laboratory diet (4 RF 21. Mucedola, Settimo Milanese, Italy) and tap water ad libitum. Feeding with LAB consortium was defined according to Butorac et al. [20]. Animal experiments were carried out in accordance with the EU Directive 2010/63/EU [40] for animal experiments, including approval by local authorities and an animal ethics committee (Law on the Welfare of Animals. NN135/06 and NN37/13; the Bioethics Committee of the Faculty of Science, University of Zagreb. Croatia (No. HR-POK-012) and in compliance with the Guide for the Care and Use of Laboratory Animals, DHHS Publ. # (NIH) 86-123.

#### 4.5.2. Bacterial 16S rRNA Sequencing and Processing Using QIIME

Faeces samples were collected from rats at the end of the study and used to purify total genomic DNA using a cDNA extraction Maxwell DNA Tissue Kit with an automated Maxwell^®^ 16 Research System instrument (Promega, Madison, WI, USA). The final equimolar pool was sequenced on the Illumina MiSeq platform. PCR reactions and 16S sequencing were performed at the Molecular Research LP (MRDNA, Shallowater, TX, USA). The MiSeq instrument (Illumina, San Diego, CA, USA) was used for sequencing the 16S amplicons following the manufacturer’s instructions at MRDNA described by Garcia-Mazcorro et al. [41] with slight modifications. Raw 16S data were obtained from Illumina’s basespace as FASTQ files and analysed using the QIIME 2 pipeline using the procedure as described in the moving pictures tutorial (https://docs.qiime2.org/2018.11/tutorials/moving-pictures/ accessed on: 15 November 2021).

### 4.6. Cheese Production

Dried fresh cows’ cheeses were produced in three replicates using fresh skimmed milk with 3.2% (*w*/*v*) milk fat (Veronika, Desinić, Croatia). Control cheese was produced using a commercial starter culture DSM CT-203 containing *Lactococcus lactis* subsp. *lactis, Lactococcus lactis* subsp. *cremoris, Lactococcus lactis* subsp. *lactis* biovar *diacetylactis* and *Leuconostoc* spp. After optimizing the conditions for the production of cheese, overnight cultures of selected LAB strains were centrifuged for 10 min at 4200 rpm, and the bacterial pellet was washed twice with sterile saline. Each sample of cheese was made from 2 L of milk inoculated with 1.87 × 10^6^ (±0.86) CFU/mL of *L. brevis* D6, 2.65 × 10^6^ (±0.62) CFU/mL of *L. fermentum* D12, 1.44 × 10^6^ (±0.68) CFU/mL of *L. plantarum* D13 and of 5.22 × 10^6^ (±1.65) CFU/mL of *E. faecium* ZGZA7-10.

Produced cheeses were analysed following the standard method [42]. The amounts of fat and lactose were determined from 50 g of samples of dried fresh cheese and 50 mL of whey at the Food Control Centre (Zagreb, Croatia). The amount of fat was determined by the Soxhlet method and the amount of lactose by the gravimetric method [43]. The pH of the milk was measured using a pH meter (Lab 845, SI Analytics, Xylem, Rye Brook, NY, USA). Sensory evaluation of the produced cheese [44] and whey [45] was performed by a panel, whose five members were trained and acquainted with the composition and properties of dried fresh cheeses, as well as process parameters during the production and storage of cheeses, with over 10 years of experience. Panelists conducted a sensory evaluation of the appearance, odour and taste of dried fresh cheeses and separated whey. Additionally, the consistency and cross-section of cheese samples, as well as the color and precipitate of whey samples, were evaluated. Each attribute was scored using commonly used scales [44,45]. Finally, produced cheeses were tested for the presence of *L. monocytogenes* and *Salmonella* spp. using the standard method by enumeration on selective ChromoBio^®^ Listeria agar (Biolab, Budapest, Hungary) and XLD agar (Biolife, Milan, Italy), respectively.

### 4.7. Analysis of Biopeptides and Proteolytic System

#### 4.7.1. Inoculum for Peptide Determination

Strains D6, D12, D13 and ZGZA7-10 were inoculated into 10 mL of MRS broth and incubated overnight anaerobically at 37 °C. Overnight cultures were centrifuged for 10 min at 4200 rpm and 4 °C. Four percent of the inoculum was added to 50 mL of milk in three samples, and incubated at 37 °C for 24 h and 48 h, respectively. The pH value after the growth of respective strains in skimmed milk was determined. Cheese and whey samples were analysed as stated above (Section 4.6.).

#### 4.7.2. Analysis of Proteolytic Enzymes by One-Dimensional Electrophoresis Coupled to MALDI-TOF/TOF

An overnight culture was propagated in 400 mL of MRS broth at 37 °C and was centrifuged for 10 min at 4200 rpm and 4 °C. Protein extraction was performed by the procedures described in the ReadyPrep Total Protein Extraction Kit (BioRad Laboratories, Berkeley, CA, USA) according to the manufacturer’s instructions. Protein extracts were separated by SDS-PAGE electrophoresis. In-gel digestion was performed according to Shevchenko et al. [46]. Dried peptides were dissolved in 10 µL of 0.1% aqueous solution of trifluoroacetic acid (TFA, Sigma-Aldrich, Merck, St. Louis, MO, USA). The peptides were then separated by NanoLC system Dionex Ultimate 3000 RSLCnano (Thermo Fisher Scientific, Waltham, MA, USA) coupled to Proteineer fcII spotter (Bruker, Bremen, Germany). Chromatographic separation was performed on a column Acclaim PepMap 100 C18 3 µm, 100 Å, 75 µm i.d. × 15 cm (Thermo Fisher Scientific, Waltham, MA, USA) at 40 °C. The flow rate was 0.3 µL/min, and the injection volume was set to 1 µL. Mobile phase A consisted of a 0.1% aqueous solution of TFA (*v*/*v*) and mobile phase B 0.1% of TFA in acetonitrile (ACN, *v*/*v*). Gradient elution was programmed to increase over 70 min with Solvent B from 2% to 90% and then to condition the column back to the initial conditions. The total run time was 75 min. The detection wavelength of the detector was set at 214 nm. The spotter flow rate was set to 100 µL/h (1.4 mg CHCA matrix dissolved in 1 mL of 50% acetonitrile aqueous solution). The total number of collected fractions was 192. Mass spectrometry acquisition (MS) was performed with an Autoflex speed MALDI TOF/TOF analyzer (Bruker, Bremen, Germany). Mass spectra were obtained in positive ion reflectron mode, in a mass range m/z 700–4000. Tandem MS analysis (MS/MS) was done with the following parameters: signal-to-noise (S/N) threshold 10, 100 ppm mass tolerance between compounds, merging compounds separated by less than 6 fractions and 5.0 Da as minimal mass distance to co-eluting compounds. SwissProt and NCBI database searches were performed by ProteinProspector MS-Tag with the following parameters: one trypsin miss cleavage and precursor mass tolerance 100 ppm.

#### 4.7.3. Identification of Bioactive Peptides

The lyophilized samples were dissolved in milli-Q water (Sigma-Aldrich, Merck, St. Louis, MO, USA) to a final concentration of 1 mg/mL and filtered through a 0.2 µm pore filter (Sigma-Aldrich, Merck, St. Louis, MO, USA). Peptide separation and MS acquisition were performed as described in Section 4.7.2. A SwissProt database search, taxonomy mammals, was performed by ProteinScape version 3.0 (Bruker, Bremen, Germany) with the following parameters: unspecified cleavage, oxidation on methionine, histidine and tryptophan, deamination of asparagine and glutamine as variable modifications with precursor mass tolerance 100 ppm. Identified peptides were searched compared with Milk Bioactive Peptide Database [47] to identify known and potential bioactive peptides.

#### 4.7.4. Relative Quantification of Bioactive Peptides

After identification of bioactive peptides, Skyline software version 21.1.0.278 [48] was used for multiple reaction monitoring (MRM) method optimization. Method development and sample analysis was performed by 6460 TripleQuad LC/MS system (Agilent Technologies, Santa Clara, CA, USA) equipped with an electrospray ionization source. Ion source and chromatographic parameters were set as described previously [49]. Briefly, the instrument was operated in positive electrospray ionization mode (ESI+). Acquity UPLC BEH C18 (2.1 × 150 mm, 1.7 µm) column from Waters Corporation (Milford, CT, USA) was used for chromatographic separation. The flow rate was set to 0.3 mL/min. Mobile phase A consisted of 0.1% of aqueous formic acid (*v*/*v*), and mobile phase B 0.1% of formic acid in ACN (*v*/*v*). The injection volume was 10 µL. Ion source parameters were set as follows: capillary voltage 3.5 kV, gas temperature 300 °C, gas flow 7 L/min, nebulizer 40 psi, sheath gas temperature 300 °C and sheath gas flow 9 L/min. Peak identification and spectral analysis were performed using MassHunter Workstation software (Agilent Technologies, Santa Clara, CA, USA). Standard peptides used for MRM method development were purchased from Thermo Scientific. The final MRM transition list is shown in Supplementary Materials Table S2. All experiments were performed independently in at least three biological replicates.

### 4.8. Statistical Analysis

Statistical analyses were performed with the Statistical Computation Web Site, VassarStats“ (http://vassarstats.net/test accessed on 9 November 2021). Statistical significance was appraised by one-way analysis of variance, and pairwise differences between the means of groups were determined by the Tukey HSD test for post analysis of variance pairwise comparisons. Statistical differences between groups were considered significant when *p* values were less than 0.1.

## 5. Conclusions

A consortium of three well-defined *Lactobacillus* and one *Enterococcus*, which impacted the alteration of intestinal microbiota in vivo, was designed as a functional dairy starter culture. Implementation of the LAB consortium affected the content of the biopeptides, favouring the release of β-CN-derived peptides through proteolytic activity. The specific functionalities of the particular probiotic strains of the LAB consortium may contribute to the added value of the final fermented product. Further studies are necessary to confirm the relationship between the addition of the newly designed probiotic culture consisting of autochthonous LAB strains in the manufacture of cheese and functional physiological effects associated with its consumption.

## Figures and Tables

**Table 1:** (**a**) Proteolytic activity determined by Anson’s method and lactate, acetate, diacetyl and lactose concentrations after overnight growth of single strains of the lactic acid bacteria (LAB) consortium in skimmed milk. (**b**) pH values and lactic acid (g/L) of the culture supernatants of single strains of the LAB consortium at 0, 6, 24 and 48 h of cultivation in skimmed milk with or without NaCl addition in concentrations of 2, 4 and 6% (*w*/*v*), as well as LAB counts expressed as the difference of logarithmic values during cultivation (Δlog CFU/mL).

**(a)**											
**LAB Strain**		**Proteolytic Activity (U/mL)**			**Acetate (mg/mL)**		**Lactate (mg/mL)**	**Diacetyl (mg/mL)**		**Lactose (mg/mL)**	
*L. plantarum* D13		1.03 ± 0.32 ^b^			2.795 ± 0.025 ^a^		4.9 ± 0 ^c^	n.d. *		51 ± 0 ^a^	
*L. brevis* D6		1.22 ± 0.18 ^b^			2.09 ± 0.16 ^c^		3.7 ± 0 ^d^	n.d.		51.0 ± 1.0 ^a^	
*L. fermentum* D12		0.79 ± 0.36 ^b^			2.310 ± 0.010 ^b^		5.850 ± 0.050 ^b^	n.d.		47.50 ± 0.50 ^b^	
*E. faecium* ZGZA7-10 **		3.52 ± 0.20 ^a^			1.420 ± 0.040 ^d^		7.150 ± 0.050 ^a^	n.d.		46.50 ± 0.50 ^b^	
**(b)**											
**Incubation Time**	**pH**				**Lactic Acid (g/L)**				**Δlog CFU/mL**		
	**0 h**	**6 h**	**24 h**	**48 h**	**0 h**	**6 h**	**24 h**	**48 h**	**6 h**	**24 h**	**48 h**
***L. plantarum* D13**											
Skimmed milk	6.58 ± 0.17	6.43 ± 0.20	5.43 ± 0.43	4.60 ± 0.67	n.d.	1.20 ± 1.04	3.30 ± 1.37	6.3 ± 3.1	0.04 ± 0.02	0.39 ± 0.35	0.080 ± 0.300
2% NaCl	6.37 ± 0.16	6.25 ± 0.22	6.01 ± 0.31	5.11 ± 0.68	n.d.	1.20 ± 0.26	1.80 ± 0.90	4.2 ± 2.1	0.20 ± 0.06	0.28 ± 0.16	−0.13 ± 0.24
4% NaCl	6.36 ± 0.25	6.21 ± 0.24	6.01 ± 0.21	5.12 ± 0.48	n.d.	1.20 ± 0.26	1.65 ± 0.69	4.2 ± 2.1	0.31 ± 0.04	0.330 ± 0.090	−0.10 ± 0.25
6% NaCl	6.31 ± 0.28	6.15 ± 0.21	6.010 ± 0.070	5.61 ± 0.22	n.d.	1.05 ± 0.26	1.35 ± 0.45	3.0 ± 1.8	0.12 ± 0.12	−0.86 ± 0.23	−0.940 ± 0.040
***L. brevis* D6**											
Skimmed milk	6.57 ± 0.12	6.35 ± 0.21	5.87 ± 0.57	4.48 ± 0.30	n.d.	1.2 ± 1.0	3.0 ± 2.8	5.7 ± 3.4	0.100 ± 0.070	0.14 ± 0.22	0.610 ± 0.050
2% NaCl	6.42 ± 0.20	6.28 ± 0.25	5.99 ± 0.34	5.2 ± 1.1	n.d.	1.2 ± 1.0	1.6 ± 1.4	4.2 ± 2.9	0.020 ± 0.090	−0.100 ± 0.020	−0.610 ± 0.060
4% NaCl	6.38 ± 0.24	6.27 ± 0.29	6.08 ± 0.27	5.38 ± 0.90	n.d.	1.05 ± 0.94	1.4 ± 1.2	3.9 ± 2.6	0.020 ± 0.030	−0.37 ± 0.15	−0.75 ± 0.24
6% NaCl	6.31 ± 0.24	6.22 ± 0.29	6.04 ± 0.20	5.60 ± 0.55	n.d.	1.05 ± 0.94	1.4 ± 1.2	3.3 ± 2.1	−0.09 ± 0.10	−0.13 ± 0.06	−0.81 ± 0.18
***L. fermentum* D12**											
Skimmed milk	6.62 ± 0.12	6.450 ± 0.080	5.23 ± 0.67	4.32 ± 0.31	n.d.	0.75 ± 0.69	2.70 ± 0.90	5.0 ± 3.0	0.01 ± 0.12	0.200 ± 0.090	0.57 ± 0.19
2% NaCl	6.44 ± 0.20	6.33 ± 0.19	5.65 ± 0.43	4.24 ± 0.39	n.d.	1.20 ± 0.26	2.25 ± 0.45	4.5 ± 1.6	−0.13 ± 0.18	0.40 ± 0.30	0.43 ± 0.25
4% NaCl	6.39 ± 0.21	6.29 ± 0.20	5.78 ± 0.28	4.77 ± 0.35	n.d.	0.75 ± 0.69	1.50 ± 0.52	4.2 ± 2.1	−0.03 ± 0.19	0.26 ± 0.33	0.36 ± 0.19
6% NaCl	6.31 ± 0.20	6.23 ± 0.20	6.020 ± 0.080	5.31 ± 0.12	n.d.	1.35 ± 0.45	1.65 ± 0.69	2.70 ± 0.90	0.01 ± 0.14	−0.44 ± 0.32	−0.44 ± 0.18
***E. faecium* ZGZA7-10**											
Skimmed milk	6.67 ± 0.08	6.21 ± 0.23	5.13 ± 0.34	4.63 ± 0.15	n.d.	1.50 ± 0.52	3.2 ± 1.2	5.1 ± 2.1	0.27 ± 0.20	0.520 ± 0.050	0.72 ± 0.31
2% NaCl	6.45 ± 0.13	6.14 ± 0.24	5.15 ± 0.30	4.730 ± 0.080	n.d.	1.65 ± 0.26	3.3 ± 1.4	5.1 ± 2.3	0.29 ± 0.24	0.37 ± 0.27	0.41 ± 0.36
4% NaCl	6.44 ± 0.22	6.21 ± 0.19	5.43 ± 0.36	5.03 ± 0.14	n.d.	1.05 ± 0.94	3.0 ± 1.4	4.5 ± 1.6	0.20 ± 0.30	0.28 ± 0.30	0.47 ± 0.18
6% NaCl	6.40 ± 0.22	6.22 ± 0.17	5.61 ± 0.22	5.40 ± 0.12	n.d.	1.2 ± 1.0	2.4 ± 1.4	3.0 ± 1.4	−0.24 ± 0.15	0.16 ± 0.26	−0.15 ± 0.34

* n.d.—not detected; LOD of the diacetyl by GC-FID is 100 ppm. ** Haemolytic activity on Columbia Blood Agar (Oxoid LTD, Basingstoke, UK) with 5% sheep blood was not detected. Values are mean ± standard deviations of results from three separate evaluations. ^a,b,c,d^ Values in the same column having different letters in superscript differ significantly (*p* < 0.1).

**Table 2:** Physicochemical properties of dried fresh cheese made by the LAB consortium compared to control cheese (fermented by starter culture DSM CT-203).

Parameter	Cheese Fermented by LAB Consortium	Control Cheese
pH *	4.180 ± 0.040 ^a^	4.440 ± 0.050 ^a^
Dry matter (%, *w*/*w*)	60.2 ± 2.4 ^b^	70.4 ± 1.5 ^a^
Fat (%, *w*/*w*)	19.06 ± 0.16 ^b^	26.57 ± 0.24 ^a^
Lactose (%, *w*/*w*)	4.24 ± 0.21 ^b^	5.82 ± 0.25 ^a^
Cheese yield (g)	12.5 ± 1.3 ^a^	11.98 ± 0.72 ^a^
Syneresis (L)	1.20 ± 0.15 ^a^	1.13 ± 0.24 ^a^
*Listeria* or *Salmonella* (CFU/mL)	n.d. **	n.d.

* pH value of the milk used for fermentation was 6.720 ± 0.020. ** n.d., not detected—no contamination on *Listeria* or *Salmonella.* Values are mean ± standard deviations of results from three separate evaluations. ^a,b^ Values in the same row having different letters in superscript differ significantly (*p* < 0.1).

**Table 3:** Preference test scores for dried fresh cheeses and separated whey, made by the LAB consortium and commercial starter culture (control).

Sample	Starter Culture	Appearance	Odour	Consistency	Cross Section	Color	Precipitate	Taste
Dried fresh cheese	LAB consortium	3.60 ± 0.55 ^a^	5.00 ± 0.00 ^a^	3.80 ± 0.45 ^a^	4.20 ± 0.45 ^a^	n.d.	n.d.	5.00 ± 0.00 ^a^
	Control	3.20 ± 0.45 ^a^	3.40 ± 0.55 ^b^	3.20 ± 0.45 ^a^	4.00 ± 0.71 ^a^	n.d.	n.d.	3.20 ± 0.84 ^b^
Whey	LAB consortium	4.40 ± 0.89 ^a^	4.80 ± 0.45 ^a^	n.d.	n.d.	4.80 ± 0.45 ^a^	4.60 ± 0.89 ^a^	4.80 ± 0.45 ^a^
	Control	3.80 ± 0.84 ^a^	4.00 ± 0.71 ^a^	n.d.	n.d.	4.00 ± 0.71 ^a^	3.00 ± 0.71 ^b^	3.40 ± 0.89 ^b^

n.d.—not determined. Values are mean ± standard deviations of results from three separate evaluations. ^a,b^ Values in the same column having different letters in superscript differ significantly (*p* < 0.1).

**Table 4:** The major peptide components of the water-soluble extracts of (**a**) skimmed milk inoculated with individual strains of LAB consortium; (**b**) dried fresh cheese fermented by LAB consortium, control cheese (fermented by DSM CT-203 starter culture), their separated whey, and milk before starter inoculation. Peptides were identified with LC-MALDI-TOF/TOF. Values are normalized data obtained after targeted LC-MRM-MS analysis.

**(a)**								
**Peptide Sequence/Protein Source**	***L. plantarum***		***L. brevis***		***L. fermentum***		***E. faecium***	
	D13 (24 h)	D13 (48 h)	D6 (24 h)	D6 (48 h)	D12 (24 h)	D12 (48 h)	ZGZA7-10 (24 h)	ZGZA7-10 (48 h)
WMHQPHQPLPPT/ Beta-casein	248.75± 114.43	131.14 ± 102.02	239.13 ± 98.18	168.66 ± 99.33	138.90 ± 26.54	195.07 ± 55.73	3.09 ± 2.37	n.d.
SWMHQPHQPLPPT/ Beta-casein	45.83 ± 17.58	23.53 ± 23.18	24.64 ± 8.35	16.50± 11.51	8.36 ± 3.29	55.74 ± 19.51	n.d.	n.d.
SQSKVLPVPQKAVPYPQ/ Beta-casein	n.d.	n.d.	n.d.	n.d.	n.d.	n.d.	n.d.	n.d.
YQEPVLGPVR/ Beta-casein	n.d.	n.d.	n.d.	n.d.	n.d.	n.d.	n.d.	n.d.
RDMPIQAF/ Beta-casein	n.d.	n.d.	n.d.	n.d.	n.d.	n.d.	n.d.	n.d.
HQPHQPLPPTVMFPPQ/ Beta-casein	n.d.	n.d.	n.d.	n.d.	n.d.	7.53 ± 3.01	n.d.	n.d.
TKVIPYVRYL/ Alpha-S2-casein	4.19 ± 1.48	1.17 ± 0.86	2.39 ± 2.23	2.67 ± 0.62	n.d.	n.d.	6.40 ± 1.62	4.01 ± 3.38
VLGPVRGPFP/ Beta-casein	36.96 ± 21.31	89.09 ± 123.62	39.27 ± 14.51	26.89 ± 17.50	89.95 ± 32.41	396.76 ± 109.19	1.08 ± 0.77	n.d.
WIQPKTKVIPYVRYL/Alpha-S2-casein	8.36 ± 2.16	5.92 ± 3.46	10.13 ± 3.77	6.95 ± 4.49	n.d.	n.d.	n.d.	n.d.
APSFSDIPNPIGSENSE/ Alpha-S1-casein	n.d.	n.d.	n.d.	n.d.	n.d.	n.d.	n.d.	n.d.
LVYPFPGPIHNSLPQN/ Beta-casein	15.02 ± 3.00	15.60 ± 3.33	14.13 ± 2.43	8.28 ± 8.27	10.14 ± 2.74	11.17 ± 0.85	n.d.	n.d.
LVYPFPGPIHNSLPQ/ Beta-casein	3.59 ± 2.24	2.59 ± 2.02	3.32 ± 3.76	3.54 ± 0.30	6.38 ± 7.09	10.58 ± 3.18	n.d.	n.d.
VYPFPGPIPN/ Beta-casein	n.d.	n.d.	1.27 ± 0.81	n.d.	n.d.	n.d.	n.d.	n.d.
QEPVLGPVRGPFPIIV/ Beta-casein	9.70 ± 1.41	4.61 ± 3.60	5.65 ± 2.93	3.32 ± 2.15	2.11 ± 1.94	5.62 ± 0.54	2.64 ± 0.67	1.65 ± 1.39
YQEPVLGPVRGPFPIIV/ Beta-casein	95.53 ± 17.96	74.71 ± 32.21	49.54 ± 14.87	26.41 ± 20.03	52.71 ± 31.15	65.61 ± 8.68	28.48 ± 7.49	17.98 ± 14.83
FVAPFPEVFG/ Alpha-S1-casein	n.d.	1.03 ± 0.61	n.d.	n.d.	n.d.	n.d.	n.d.	n.d.
LYQEPVLGPVRGPFPIIV/ Beta-casein	27.31 ± 13.59	7.24 ± 4.11	20.43 ± 17.83	12.88 ± 10.89	11.61 ± 9.29	12.41 ± 2.45	3.62 ± 3.06	3.34 ± 0.40
LLYQEPVLGPVRGPFPIIV/ Beta-casein	3.22 ± 1.82	n.d.	3.20 ± 4.15	n.d.	1.05 ± 0.73	1.07 ± 0.13	1.13 ± 1.59	n.d.
**(b)**								
**Peptide Sequence/** **Protein Source**	**RT**	**Control Cheese**		**Control Whey**	**Cheese with LAB Consortium**	**Whey of LAB Consortium**		**Control**
WMHQPHQPLPPT/ Beta-casein	8.953	1.67 ± 0.72		n.d.	13.08 ± 0.89	1.14 ± 0.11		n.d.
SWMHQPHQPLPPT/ Beta-casein	9.446	20.41 ± 2.79		46.92 ± 3.82	132.39 ± 18.65	22.10 ± 8.07		n.d.
SQSKVLPVPQKAVPYPQ/ Beta-casein	10.069	56.48 ± 28.21		46.01 ± 7.46	90.90 ± 3.32	35.34 ± 7.67		n.d.
YQEPVLGPVR/ Beta-casein	10.210	2.84 ± 0.95		n.d.	7.69 ± 0.29	n.d.		n.d.
RDMPIQAF/ Beta-casein	11.373	51.08 ± 12.79		25.32 ± 0.31	108.89 ± 5.37	7.70 ± 0.21		n.d.
HQPHQPLPPTVMFPPQ/ Beta-casein	11.582	n.d.		n.d.	9.55 ± 1.80	4.25 ± 0.75		n.d.
TKVIPYVRYL/ Alpha-S2-casein	11.723	49.02 ± 12.40		69.00 ± 11.30	35.52 ± 6.36	12.59 ± 5.62		40.70 ± 6.50
VLGPVRGPFP/ Beta-casein	12.083	n.d.		n.d.	n.d.	n.d.		n.d.
WIQPKTKVIPYVRYL/ Alpha-S2-casein	12.172	n.d.		n.d.	n.d.	n.d.		n.d.
APSFSDIPNPIGSENSE/ Alpha-S1-casein	12.506	6.54 ± 0.40		n.d.	20.89 ± 0.21	n.d.		n.d.
LVYPFPGPIHNSLPQN/ Beta-casein	12.917	n.d.		n.d.	1.74 ± 2.42	n.d.		n.d.
LVYPFPGPIHNSLPQ/ Beta-casein	13.279	2.77 ± 0.38		5.94 ± 0.78	1.49 ± 2.08	1.24 ± 0.36		n.d.
VYPFPGPIPN/ Beta-casein	13.373	1.69 ± 0.67		1.85 ± 0.53	7.66 ± 0.33	n.d		n.d.
QEPVLGPVRGPFPIIV/ Beta-casein	15.696	19.27 ± 1.83		125.57 ± 19.77	25.93 ± 2.05	77.66 ± 24.10		n.d.
YQEPVLGPVRGPFPIIV/ Beta-casein	15.949	1432.50 ± 228.82		361.51 ± 33.00	682.92 ± 57.39	97.39 ± 30.35		11.25 ± 6.08
FVAPFPEVFG/ Alpha-S1-casein	16.093	37.60 ± 3.70		n.d.	35.79 ± 0.70	n.d.		n.d.
LYQEPVLGPVRGPFPIIV/ Beta-casein	16.250	47.63 ± 10.13		6.54 ± 1.20	20.24 ± 0.44	2.64 ± 0.32		5.82 ± 2.54
LLYQEPVLGPVRGPFPIIV/Beta-casein	16.743	9.05 ± 3.15		8.37 ± 1.31	n.d.	n.d.		n.d.

n.d.—not detected.

## Data Availability

Not applicable.

## Supplementary Materials

The following are available online. Table S1: Identification of the putative proteins of *E. faecium* ZGZA7-10 proteolytic system using LC-MALDI-TOF/TOF analysis and MS-Tag browser via: (a) SwissProt and (b) NCBI database. Table S2: Transition list used for peptide relative quantification.

## Sample Availability

Samples of the compounds are not available from the authors.
