# Supplementary material for: A Lactic Acid Bacteria Consortium Impacted the Content of Casein-Derived Biopeptides in Dried Fresh Cheese
Source: Molecules. 2021 Dec 28;27(1):160. doi: 10.3390/molecules27010160 (PMC8746304; doi:10.3390/molecules27010160)
Supplement: Supplementary file 1 [file molecules-27-00160-s001.zip › molecules-1520309-supplementary.pdf]

Supplementary Table S1. Identification of the putative proteins of *E. faecium* ZGZA7-10 proteolytic system using LC-MALDI-TOF/TOF analysis and MS-Tag browser via: **(a)** SwissProt and **(b)** NCBI database.

(a)

Amino acid sequence	MH+ (Da)	Protein MW (Da)/pI	Score	Putative protein name
		PROTEASES		
(R)VELAKLSVDDFVR(I)	1490.8	52,349/5.80	12.0	ATP-dependent protease ATPase subunit HslU
(K)ILDADHYGLEKVK(E)	1500.8	90,144/8.0	3.20	Lon protease
(K)AAVDYSIQYIPQR(S)	1523.8	77,430/5.60	9.90	Probable ATP-dependent Clp protease ATP-binding subunit
(R)SEKPQLRATTVLGVIR(N)	1768.0	19,727/6.80	5.30	ATP-dependent protease subunit HslV
(K)FKVLSQSEVDDLMTEK(S)	1868.9	450,063/5.50	10.90	Adenosine monophosphate-protein transferase and cysteine protease IbpA
(K)VPFAIADATTLTEAGYVGEDVENILLK(L)	2849.5	45,478/7.50	14.80	ATP-dependent Clp protease ATP-binding subunit ClpX
R.DFNEALVHQVVVAYAR.Q	2030.0	86,000/6.55	84.25	ATP-dependent Clp protease ATP-binding subunit ClpL

(b)

Amino acid sequence	MH+ (Da)	Protein MW (Da)/pI	Score	Putative protein name
		PEPTIDASES		
(R)DNAGTVK(G)	704.4	175,950/9.4	2.1	peptidase
(R)LESANAK(S)	732.4	69,508/8.6	18.6	peptidase
(R)VSGPVIR(M)	727.4	79,499/6.7	13.7	peptidase C39
(K)VSVLYR(K)	736.4	50,629/6.0	15.8	leucyl aminopeptidase
(K)VSVLYR(K)	736.4	31,980/7.1	15.8	cytosol aminopeptidase family protein. partial
		TRANSPORT SYSTEM		
(K)ADVLYR(C)	736.4	402,70/5.3	14.7	phosphoserine aminotransferase
(R)GEVLYR(G)	736.4	65,183/9.3	14.5	amino acid composite ATP-binding transmembrane ABC transporter protein
(R)ASAATSAR(N)	734.4	46,252/9.9	12.8	high-affinity branched-chain amino acid transport system permease LivM

Supplementary Table S2. Transition list used for peptide relative quantification.

Peptide	Precursor Ion	Product Ion	Dwell	Fragmentor Voltage	Collision Energy	Cell Accelerator Voltage	Polarity
QEPVLGPVRGPFPIIV	859.50	730.95	20	130	27.6	4	Positive
		567.31	20	130	27.6	4	Positive
		744.42	20	130	27.6	4	Positive
		800.96	20	130	27.6	4	Positive
LLYQEPVLGPVRGPFPIIV	703.08	390.24	20	130	20.5	4	Positive
		518.30	20	130	20.5	4	Positive
		647.34	20	130	20.5	4	Positive
		843.46	20	130	20.5	4	Positive
YQEPVLGPVRGPFPIIV	627.69	720.89	20	130	17.8	4	Positive
		769.41	20	130	17.8	4	Positive
		825.95	20	130	17.8	4	Positive
		882.50	20	130	17.8	4	Positive
LYQEPVLGPVRGPFPIIV	665.39	655.37	20	130	19.2	4	Positive
		777.43	20	130	19.2	4	Positive
		825.95	20	130	19.2	4	Positive
		882.50	20	130	19.2	4	Positive
WMHQPHQPLPPT	490.24	473.21	20	130	12.8	4	Positive
		521.74	20	130	12.8	4	Positive
		578.28	20	130	12.8	4	Positive
		626.81	20	130	12.8	4	Positive
VLGPVRGPFP	519.81	470.27	20	130	17.1	4	Positive
		413.73	20	130	17.1	4	Positive
		385.22	20	130	17.1	4	Positive
		462.28	20	130	17.1	4	Positive
WIQPKTKVIPYVRYL	476.78	810.45	20	130	12.4	4	Positive
		713.40	20	130	12.4	4	Positive
		550.33	20	130	12.4	4	Positive
		451.27	20	130	12.4	4	Positive

LVYPFPGPIHNSLPQN	896.97	790.90	20	130	28.8	4	Positive
		709.36	20	130	28.8	4	Positive
		587.30	20	130	28.8	4	Positive
LVYPFPGPIHNSLPQ	839.95	733.87	20	130	27.0	4	Positive
		652.34	20	130	27.0	4	Positive
		530.28	20	130	27.0	4	Positive
HQPHQPLPPTVMFPPQ	617.65	616.83	20	130	17.4	4	Positive
		682.35	20	130	17.4	4	Positive
		755.88	20	130	17.4	4	Positive
		804.41	20	130	17.4	4	Positive
SWMHQPHQPLPPT	519.25	516.73	20	130	13.9	4	Positive
		565.25	20	130	13.9	4	Positive
		621.80	20	130	13.9	4	Positive
		670.32	20	130	13.9	4	Positive
FVAPFPEVFG	555.29	548.27	20	130	18.2	4	Positive
		562.30	20	130	18.2	4	Positive
		788.40	20	130	18.2	4	Positive
		887.47	20	130	18.2	4	Positive
APSFSDIPNPIGSENSE	880.90	832.37	20	130	28.3	4	Positive
		622.23	20	130	28.3	4	Positive
		605.26	20	130	28.3	4	Positive
		718.34	20	130	28.3	4	Positive
RDMPIQAF	489.25	403.18	20	130	16.2	4	Positive
		613.31	20	130	16.2	4	Positive
		741.37	20	130	16.2	4	Positive
		812.41	20	130	16.2	4	Positive
VYPFPGPIPN	550.79	838.45	20	130	18.1	4	Positive
		594.32	20	130	18.1	4	Positive
		360.19	20	130	18.1	4	Positive
		871.47	20	130	18.1	4	Positive

SQSKVLPVPQKAVPYPQ	622.69	514.28	20	130	17.6	4	Positive
		370.72	20	130	17.6	4	Positive
		681.91	20	130	17.6	4	Positive
		811.97	20	130	17.6	4	Positive
YQEPVLGPVR	579.32	866.51	20	130	19.0	4	Positive
		737.47	20	130	19.0	4	Positive
		428.26	20	130	19.0	4	Positive
		421.17	20	130	19.0	4	Positive
TKVIPYVRYL	417.92	713.40	20	130	10.2	4	Positive
		550.33	20	130	10.2	4	Positive
		451.27	20	130	10.2	4	Positive
		442.30	20	130	10.2	4	Positive
